# Physical Activity–Sedentary Behaviour Profiles and Associated Lifestyle Characteristics in Adolescents: A Cross-Sectional Study

**DOI:** 10.3390/nu18172798

**Published:** 2026-08-27

**Authors:** Mariola Wojciechowska, Elżbieta Cieśla, Arkadiusz Łukasz Żurawski, Anna Zmyślna, Dorota Kozieł

**Affiliations:** 1 Department of Social Problems and Social Work, Institute of Pedagogy, Faculty of Pedagogy and Psychology, Jan Kochanowski University in Kielce, Krakowska 11, 25-029 Kielce, Poland; mariola.wojciechowska@ujk.edu.pl; 2 Department of Nutrition and Dietetics, Institute of Health Sciences, Faculty of Health Sciences, Collegium Medicum, Jan Kochanowski University in Kielce, al. IX Wieków Kielc 19A, 25-516 Kielce, Poland; elzbieta.ciesla@ujk.edu.pl; 3 Department of Physiotherapy, Institute of Health Sciences, Faculty of Health Sciences, Collegium Medicum, Jan Kochanowski University in Kielce, al. IX Wieków Kielc 19A, 25-516 Kielce, Poland; anna.zmyslna@ujk.edu.pl; 4 Department of Nursing and Other Health Professions, School of Public Health, Centre of Postgraduate Medical Education, Marymoncka 99/103, 01-813 Warsaw, Poland; dorota.koziel@cmkp.edu.pl

**Keywords:** adolescents, physical activity, sedentary behaviour, movement behaviour, sleep duration, eating regularity, lifestyle profiles, cluster analysis

## Abstract

**Background/Objectives:** Physical activity and sedentary behaviour are related but distinct components of adolescent movement behaviour and may combine into heterogeneous lifestyle profiles. This study aimed to identify empirically derived physical activity–sedentary behaviour profiles and to examine sociodemographic and lifestyle factors associated with profile membership. **Methods:** This cross-sectional, school-based study included 3307 Polish adolescents aged 15–17 years (52.22% girls; mean age, 15.89 ± 0.71 years). Weekly sedentary time and walking, moderate-intensity, and vigorous-intensity physical activity were assessed using the IPAQ-LF, reprocessed using predefined truncation procedures, standardised using z-scores, and entered into k-means clustering. Two- and three-cluster solutions were evaluated. Separate binary logistic regression models examined crude and sex- and school-type-adjusted associations between candidate lifestyle correlates and profile membership. **Results:** Although the two-cluster solution had a higher average silhouette coefficient (0.36 vs. 0.28), the three-cluster solution was retained because it provided a more differentiated and substantively interpretable representation of movement-behaviour patterns. The profiles comprised a walking-dominant activity profile (C1; *n* = 645; 19.50%), a high moderate-to-vigorous activity/lower sedentary profile (C2; *n* = 354; 10.71%), and a lower physical activity profile (C3; *n* = 2308; 69.80%). C3 had substantially lower walking, moderate-intensity, and vigorous-intensity activity than C1, while sedentary time was similar in magnitude between these profiles. After adjustment for sex and school type, C2 membership was associated with irregular eating patterns (OR = 1.73, 95% CI: 1.05–2.84), school-day sleep ≥ 9 h/day (OR = 1.98, 95% CI: 1.11–3.54), weekend sleep < 7 h/day (OR = 1.70, 95% CI: 1.02–2.82), and weekend sleep ≥ 9 h/day (OR = 1.59, 95% CI: 1.16–2.16). No lifestyle candidate correlate reached statistical significance in the adjusted C3-versus-C1 analyses. **Conclusions:** The identified profiles support viewing physical activity and sedentary behaviour as related but non-equivalent dimensions of adolescent movement behaviour. Lower physical activity did not necessarily coincide with higher sedentary time, while selected sleep-duration and eating-regularity characteristics were associated with the high moderate-to-vigorous activity/lower sedentary profile. These findings are exploratory and warrant confirmation in longitudinal studies using objective measures.

## 1. Introduction

Adolescence is a critical developmental period during which health-related behaviours become established and may persist into adulthood. Physical activity and sedentary behaviour are central components of adolescent health because they are modifiable and are directly addressed in international public health recommendations for young people [1]. Higher sedentary behaviour in school-aged children and adolescents has been associated with less favourable health indicators, although the strength of these associations varies according to the type of sedentary behaviour and the health outcome considered [2,3]. At the same time, physical activity and sedentary behaviour do not occur in isolation. They are embedded within broader lifestyle patterns that may also include sleep, screen exposure, dietary behaviours, energy drink consumption, alcohol use and other health-related behaviours.

Previous research has increasingly shown that adolescent lifestyle behaviours tend to cluster into distinct behavioural configurations. Reviews of studies in children and adolescents indicate that physical activity, sedentary behaviour and dietary behaviours commonly form healthy, unhealthy and mixed profiles [4,5]. Activity-related typologies in youth also include active, inactive, sedentary and mixed profiles, suggesting that physical activity and sedentary behaviour should be interpreted as part of broader behavioural contexts rather than as isolated exposures [6]. Person-oriented methods, including cluster analysis and latent class analysis, are particularly useful in this setting because they can identify subgroups of adolescents who share similar behavioural configurations [7,8]. However, identifying behavioural phenotypes is only one step. A further question is which sociodemographic and lifestyle factors independently differentiate these phenotypes.

Several adolescent studies have identified lifestyle clusters combining movement behaviours with diet, sleep and screen-related behaviours. In the HELENA study, European adolescents were grouped into clusters based on physical activity, sedentary behaviour and dietary patterns, and cluster membership differed according to sex and parental education [9]. In the UP&DOWN Study, lifestyle clusters involving physical activity, screen time, diet and sleep were associated with health-related physical fitness [10]. In a latent class analysis of US adolescents, classes based on physical activity and screen-based sedentary behaviour were associated with sleep duration, with more favourable sleep patterns observed in the high physical activity/low screen-based sedentary behaviour class [11]. These findings indicate that movement-behaviour phenotypes may be linked with other lifestyle domains, but they do not fully clarify whether the correlates of high physical activity and high sedentary behaviour phenotypes are simply opposite or qualitatively different.

This distinction is important because high physical activity should not automatically be interpreted as a uniformly healthy lifestyle profile. Previous person-oriented studies have shown that protective and risk-related behaviours may coexist within the same adolescent subgroup. Flemish youth research identified a “sporty media-oriented mixed eaters” cluster, showing that sport-related behaviour may coexist with media use and mixed eating patterns [12]. Among Toronto adolescents, an “active, high screen-time users” cluster showed that physically active adolescents may still have high screen exposure [13]. Similarly, studies of adolescent lifestyle clusters have identified mixed profiles combining favourable and unfavourable behaviours, including diet, physical activity, screen time and sleep [5,14]. Therefore, high physical activity and high sedentary behaviour should not be assumed to represent opposite ends of a simple healthy–unhealthy continuum.

Sleep and digital behaviours are plausible correlates of physical activity–sedentary behaviour phenotypes. Screen time has been associated with shorter sleep duration and less favourable sleep outcomes in school-aged children and adolescents [15]. Access to or use of portable screen-based media devices has also been associated with inadequate sleep quantity, poor sleep quality and daytime sleepiness [16]. Problematic internet use has been associated with sleep problems, including shorter sleep duration and poorer sleep quality [17,18]. These findings suggest that sleep duration, screen time and internet-related risk may help differentiate adolescents with high sedentary exposure from those with more favourable movement-behaviour profiles.

Dietary behaviours and risk-related behaviours may also differentiate physical activity–sedentary behaviour phenotypes. Polish research has identified prudent-active and fast-food-sedentary dietary-lifestyle patterns among teenagers, indicating that activity and diet may combine into distinct lifestyle configurations with different adiposity-related correlates [19]. Energy drink consumption may be particularly relevant in physically active adolescents. In US adolescents, sports and energy drink consumption was linked not only to higher physical activity and sport participation, but also to unhealthy beverage patterns, cigarette smoking and screen media use [20]. Recent Polish data also indicate that energy drink consumption is common among physically active adolescents participating in organised sport and varies by sex and age [21]. These findings further support the need to examine high physical activity phenotypes as potentially mixed behavioural profiles rather than assuming that they are uniformly healthy.

Despite growing evidence that physical activity, sedentary behaviour and other lifestyle behaviours cluster into heterogeneous configurations during adolescence, an important knowledge gap remains. Most person-oriented studies have focused primarily on identifying behavioural subgroups, whereas less is known about how sociodemographic, sleep, screen-related, dietary, substance-related and broader health behaviours are associated with membership in empirically derived physical activity–sedentary behaviour phenotypes. Examining these associations may help clarify whether movement-behaviour phenotypes are embedded within broader lifestyle configurations rather than representing positions along a simple healthy–unhealthy continuum. This distinction is particularly relevant because favourable and unfavourable behaviours may coexist within the same adolescent subgroup.

Accordingly, the aims of this study were (1) to identify physical activity–sedentary behaviour phenotypes among adolescents using a person-oriented clustering approach and (2) to examine crude and sex- and school-type-adjusted associations between selected sociodemographic and lifestyle factors and phenotype membership. We hypothesised that the empirically derived phenotypes would show heterogeneous patterns of associated lifestyle characteristics rather than a uniform gradient of increasingly favourable or unfavourable behaviours.

## 2. Materials and Methods

### 2.1. Study Design and Setting

This cross-sectional, school-based observational study was conducted among adolescents attending secondary schools in the Świętokrzyskie Voivodeship in south-central Poland during the 2024/2025 school year. The study formed the quantitative component of the project “Youth Lifestyles and Their Health Implications: Conclusions for Pedagogical and Psychological Support and Risk Prevention” (Polish title: “Style życia młodzieży i ich zdrowotne implikacje. Wnioski w obszarze pedagogiczno-psychologicznego wsparcia oraz profilaktyki zagrożeń”), implemented by Jan Kochanowski University in Kielce, Poland. The publication was co-financed from the state budget under the Polish Minister of Education and Science programme “Science for Society II” (project No. NdS-II/SP/0429/2023/01; funding amount and total project value: PLN 1,029,600.00).

The present analysis focused on physical activity and sedentary behaviour patterns and on sociodemographic, sleep, TV/computer screen time, dietary, substance-related, and broader health behaviour factors associated with membership in empirically derived movement-behaviour phenotypes. The study was reported in accordance with the Strengthening the Reporting of Observational Studies in Epidemiology (STROBE) recommendations for cross-sectional studies.

### 2.2. Participants, Sampling and Data Collection

The target population comprised second-year students aged 15–17 years attending general secondary schools, technical secondary schools, and stage I vocational schools in the Świętokrzyskie Voivodeship. The sampling frame was constructed using the Polish Educational Information System (System Informacji Oświatowej, SIO) and was updated using the register of educational institutions maintained by the Regional Board of Education in Kielce. Information on school type, estimated enrolment, address, district (powiat), and municipality (gmina) was used to construct the sampling frame.

From an initial pool of 253 upper secondary schools, schools for adults, schools with an estimated enrolment of fewer than 10 students, and vocational preparation schools intended for young people with multiple disabilities or moderate-to-severe intellectual disabilities were excluded (*n* = 25). Schools were then selected using stratified random sampling according to school type and location. The location strata comprised large cities (>100,000 inhabitants), medium-sized urban areas, and small towns, while school-type strata comprised general secondary, technical secondary, and stage I vocational schools. A total of 53 schools were randomly selected. If a selected school declined to participate, another school was randomly selected from the same stratum.

The target sample size was approximately 3500 students, based on an estimated regional source population of approximately 12,500 students in the relevant secondary-school cohort. The study sample comprised 3307 adolescents, including 1727 girls and 1580 boys, with a mean age of 15.89 ± 0.71 years (range, 15–17 years).

Data were collected at participating schools using anonymous, self-administered paper questionnaires. The survey package comprised standardised instruments and project-specific questions. Data used in the present study were obtained from the International Physical Activity Questionnaire–Long Form (IPAQ-LF), the KomPAN Dietary Habits and Nutrition Beliefs Questionnaire, the Problematic Internet Use Test (TPUI-22), the Health Behaviour Inventory (HBI), and questions concerning sociodemographic characteristics, anthropometric variables, smoking, alcohol consumption, and energy drink consumption. The reference period for physical activity and sedentary behaviour was the preceding seven days. Following IPAQ data-quality screening and processing, all 3307 participants were retained for the profile-based analyses.

### 2.3. Ethical Considerations

The study was approved by the Research Ethics Committee of the Faculty of Pedagogy and Psychology, Jan Kochanowski University in Kielce (Resolution No. 5/2023 of 21 December 2023). Participation was voluntary. As all participants were aged 15–17 years, written informed consent was obtained from a parent or legal guardian of every participant, and all adolescents provided assent.

Students were informed about the purpose and general procedures of the study, the confidential nature of their responses, their right to decline participation, and their right to withdraw during questionnaire completion without any adverse consequences. The questionnaires contained no direct personal identifiers, and the results were reported only in aggregate form. Paper questionnaires and consent documentation were stored separately in secured university facilities. Electronic data were stored in password-protected files or university systems accessible only to authorised members of the research team.

### 2.4. Sociodemographic and Anthropometric Variables

Sociodemographic variables included sex, age, place of residence, school type, and maternal and paternal education. Place of residence was classified as village, town, or city. Maternal and paternal education were recorded separately and classified as primary/vocational, secondary, or university education. In the regression analyses, sex and school type were included as adjustment variables, whereas place of residence and parental education were used for descriptive characterisation of the study population and were not included in the regression models.

Participants self-reported their body height and body mass; no direct anthropometric measurements were performed as part of the study. Body mass index (BMI) was calculated as body mass in kilograms divided by height in metres squared (kg/m^2^) and was used descriptively in the analyses.

### 2.5. Physical Activity and Sedentary Behaviour

Physical activity and sitting time were assessed using the International Physical Activity Questionnaire–Long Form (IPAQ-LF) [22]. The questionnaire assesses walking and moderate- and vigorous-intensity physical activity across four domains: work or study, transportation, domestic and gardening activities, and leisure-time physical activity during the preceding seven days. It also assesses sitting time on weekdays and weekend days.

Prior to the analyses, IPAQ responses were subjected to data-quality screening and processed in accordance with the IPAQ data-processing procedure. Reported durations of walking, moderate-intensity physical activity, and vigorous-intensity physical activity exceeding 180 min/day were truncated to 180 min/day.

For each physical activity item, the number of minutes per day was multiplied by the number of days per week, and values were subsequently summed across domains. Walking, moderate-intensity physical activity, and vigorous-intensity physical activity were expressed in minutes per week. Weekly sedentary time was calculated as five times the reported weekday sitting time plus two times the reported weekend-day sitting time and was expressed in minutes per week. The resulting cleaned weekly values were used in the clustering analysis.

### 2.6. Sleep Duration

Sleep duration was assessed using two items from the KomPAN questionnaire concerning the average number of hours slept per day on school days and on days off from school [23]. In accordance with the response categories used in the questionnaire, sleep duration was analysed separately for school days and days off from school and classified as <7 h/day, 7–8 h/day, or ≥9 h/day. The 7–8 h/day category was used as the reference category in the regression analyses.

### 2.7. TV/Computer Screen Time and Problematic Internet Use

TV/computer screen time was assessed using a KomPAN questionnaire item asking participants how many hours per day they usually spent watching television or using a computer [23]. Responses were classified as <2 h/day, ≥2 to <4 h/day, ≥4 to <6 h/day, or ≥6 h/day. The <2 h/day category was used as the reference category in the regression analyses. This measure referred specifically to television viewing and computer use and should not be interpreted as total screen exposure across all digital devices.

Problematic Internet use was assessed using the TPUI-22, a Polish adaptation of Young’s Internet Addiction Test [24]. The instrument consists of 22 items rated on a six-point scale from 0 (“never”) to 5 (“always”), yielding a total score ranging from 0 to 110. Based on the age-specific classification proposed for the Polish TPUI-22 [24], scores were categorised as very low (0–1 points), low (2–10 points), moderate (11–49 points), high (50–79 points), or very high (80–110 points) Internet-use-related risk.

### 2.8. Dietary and Substance-Related Behaviours

Dietary behaviours were assessed using the KomPAN questionnaire [23]. Between-meal snacking, eating meals away from home, alcohol consumption, and energy drink consumption were recorded using the following frequency categories: never, 1–3 times/month, once/week, several times/week, once/day, and several times/day.

For the present analyses, between-meal snacking and eating meals away from home were each classified as never or 1–3 times/month, once or several times/week, or once or several times/day. Regular eating patterns were classified as regular, regular only for selected meals, or irregular. Alcohol consumption and energy drink consumption were dichotomised as any reported consumption versus no reported consumption. Consequently, these variables distinguished participants reporting any consumption from those reporting none, but did not differentiate occasional from frequent consumption. Current smoking was assessed using a single self-report question and coded as yes or no. The “no reported consumption” category was used as the reference category for alcohol and energy drink consumption, and “no” was used as the reference category for current smoking.

The Pro-Healthy Diet Index (pHDI-10) and Non-Healthy Diet Index (nHDI-14) were calculated according to the KomPAN methodology [23]. Reported consumption frequencies were converted into daily frequencies using the following factors: 0 for never, 0.06 for 1–3 times/month, 0.14 for once/week, 0.50 for several times/week, 1 for once/day, and 2 for several times/day.

The pHDI-10 comprised 10 food groups considered potentially beneficial to health: wholemeal bread, coarse groats, milk, fermented milk beverages, cottage cheese, white meat, fish, legumes, fruit, and vegetables. Daily frequencies were summed and converted to a 0–100-point scale by dividing the raw score by 20 and multiplying by 100.

The nHDI-14 comprised 14 food groups considered potentially adverse to health: white bread; refined groats, pasta, and rice; fast food; fried meat- or flour-based dishes; butter; lard; yellow and processed cheese; cold cuts, sausages, and frankfurters; red meat; sweets and confectionery; canned meat products; sugar-sweetened carbonated beverages; energy drinks; and alcoholic beverages. Daily frequencies were summed and converted to a 0–100-point scale by dividing the raw score by 28 and multiplying by 100. Higher pHDI-10 and nHDI-14 scores indicate more frequent consumption of the corresponding pro-healthy and non-healthy food groups, respectively.

### 2.9. Health Behaviour Inventory

General health behaviours were assessed using the Health Behaviour Inventory (HBI) [25]. The instrument comprises 24 items rated from 1 (“almost never”) to 5 (“almost always”) and covers four domains: nutritional behaviours, preventive behaviours, positive mental attitude, and health practices. Each domain contains six items. Domain scores were calculated by summing the relevant items, resulting in a possible score of 6–30 for each domain, with higher scores indicating more frequent self-reported health-promoting behaviours. In the present analyses, the four domain scores were treated as continuous variables and were evaluated separately as candidate correlates of phenotype membership.

### 2.10. Derivation of Physical Activity–Sedentary Behaviour Phenotypes

Physical activity–sedentary behaviour phenotypes were derived using k-means clustering. The four input variables were weekly sedentary time, walking physical activity, moderate-intensity physical activity, and vigorous-intensity physical activity. Prior to clustering, the cleaned weekly values of all four variables were standardised using z-score transformation so that the variables were represented on a common standardised scale. The clustering analysis was performed using TIBCO Statistica version 13.3.

K-means clustering was selected as an exploratory person-oriented approach to partition participants according to similarities in their profiles across the four continuous movement-behaviour variables. This distance-based approach provides a transparent classification of participants into mutually exclusive groups and was considered appropriate for the exploratory objective of identifying movement-behaviour phenotypes. Latent profile analysis represents an alternative model-based approach; however, the present study retained k-means clustering, with standardisation of the input variables applied to reduce the influence of differences in their numerical dispersion.

Two- and three-cluster solutions were examined. The number of clusters was evaluated using both the average silhouette coefficient and substantive interpretability of the resulting movement-behaviour profiles. The final cluster solution and its characteristics are reported in the Results section.

### 2.11. Candidate Correlates and Regression Outcome

The outcome in the logistic regression analyses was membership in the physical activity–sedentary behaviour phenotypes. Two pairwise contrasts were analysed separately: C2 versus C1 and C3 versus C1.

Candidate correlates were selected based on their conceptual relevance to adolescent lifestyle and health behaviours and comprised school-day and weekend sleep duration, TV/computer screen time, Internet-use-related risk, between-meal snacking, eating meals away from home, regular eating patterns, the Pro-Healthy Diet Index (pHDI-10), the Non-Healthy Diet Index (nHDI-14), current smoking, alcohol consumption, energy drink consumption, and the four Health Behaviour Inventory domains: nutritional behaviours, preventive behaviours, positive mental attitude, and health practices.

Sex and school type were included as adjustment variables in the adjusted regression models. Age, BMI, place of residence, and maternal and paternal education were used for descriptive characterisation of the study population and were not included in the regression analyses. The variable indicating compliance with the World Health Organisation physical activity recommendation was excluded from the regression analyses because it was derived from the same physical activity components used to construct the phenotype outcome.

Detailed operational definitions and coding are provided in Table S1.

### 2.12. Statistical Analysis

Categorical variables were summarised as numbers and percentages. Percentages were calculated using the number of participants with available data for the respective variable as the denominator, unless otherwise specified. Continuous variables were summarised using means and standard deviations and, where appropriate, medians and interquartile ranges.

Descriptive differences across the three physical activity–sedentary behaviour phenotypes were assessed using Pearson’s chi-square test for categorical variables and the Kruskal–Wallis test for continuous variables.

Associations with phenotype membership were examined using separate binary logistic regression analyses for C2 versus C1 and C3 versus C1. For each candidate correlate, an unadjusted model and an adjusted model including sex and school type as covariates were estimated. Each candidate correlate was evaluated in a separate regression model; therefore, no *p*-value-based pre-screening, stepwise variable-selection procedure, or simultaneous inclusion of multiple lifestyle correlates in a single model was applied. Continuous indices, including the dietary indices and Health Behaviour Inventory domain scores, were entered as linear continuous variables. Results are reported as odds ratios (ORs) with 95% confidence intervals (95% CIs) and *p*-values. Separate binary logistic models were used because the inferential objective focused on two prespecified profile contrasts, C2 versus C1 and C3 versus C1, rather than on an omnibus multinomial association across all three outcome categories.

Reference categories were defined a priori. Girls were the reference category for sex, and general secondary school was the reference category for school type. The reference categories for the remaining categorical variables were low/very low for Internet-use-related risk, <2 h/day for TV/computer screen time, regular eating for regular eating patterns, never or 1–3 times/month for between-meal snacking and eating meals away from home, 7–8 h/day for school-day and weekend sleep duration, and no for current smoking, reported alcohol consumption, and reported energy drink consumption.

Because the nHDI-14 includes alcoholic beverages and energy drinks, and because the HBI nutritional-behaviour domain overlaps conceptually with dietary indices and individual dietary behaviours, these variables were evaluated in separate regression models rather than entered simultaneously into the same model. This modelling strategy reduced the risk of statistical collinearity arising from simultaneous inclusion of conceptually overlapping measures.

All statistical analyses were performed using TIBCO Statistica version 13.3. Detailed operational definitions and coding are provided in Table S1.

## 3. Results

### 3.1. Study Sample and Data Availability

The study included 3307 adolescents, comprising 1727 girls (52.22%) and 1580 boys (47.78%). The mean age was 15.89 ± 0.71 years (range, 15–17 years). Body mass index data were available for 2884 participants, with a mean BMI of 21.58 ± 4.69 kg/m^2^; BMI data were missing for 423 participants (12.79%).

Among participants with available data, current smoking was reported by 759 of 3130 adolescents (24.25%), any alcohol consumption by 1219 of 3206 (38.02%), and any energy drink consumption by 1745 of 3206 (54.43%). School-day sleep duration of <7 h/day was reported by 1385 of 3122 participants (44.36%), whereas weekend sleep duration of ≥9 h/day was reported by 1722 of 3118 (55.23%). TV/computer screen time of ≥6 h/day was reported by 591 of 3140 adolescents (18.82%), and high or very high Internet-use-related risk was observed in 1795 of 3307 participants (54.28%). Characteristics of the study sample and data availability are presented in Table 1.

### 3.2. Physical Activity–Sedentary Behaviour Profiles

K-means clustering was performed using standardised values of weekly sedentary time, walking physical activity, moderate-intensity physical activity, and vigorous-intensity physical activity. Both two- and three-cluster solutions were examined. The average silhouette coefficient was higher for the two-cluster solution (0.36) than for the three-cluster solution (0.28). Nevertheless, the three-cluster solution was retained because it provided a more differentiated and substantively interpretable representation of the movement-behaviour patterns relevant to the study objective.

The retained solution comprised C1, the walking-dominant activity profile (*n* = 645; 19.50%); C2, the high moderate-to-vigorous activity/lower sedentary profile (*n* = 354; 10.71%); and C3, the lower physical activity profile (*n* = 2308; 69.80%). Global Kruskal–Wallis tests indicated differences across the three profiles for each of the four clustering variables (all *p* = 0.001).

C1 showed the highest observed mean walking physical activity (1848.20 ± 831.61 min/week), together with relatively high moderate-intensity physical activity (1714.70 ± 819.06 min/week) and lower vigorous-intensity activity than C2 (507.98 ± 229.37 min/week). Mean sedentary time in C1 was 2785.78 ± 1354.91 min/week.

C2 showed the lowest observed mean sedentary time (2297.94 ± 383.83 min/week) and the highest observed mean levels of moderate-intensity (1951.07 ± 1233.05 min/week) and vigorous-intensity physical activity (1425.51 ± 383.83 min/week). Mean walking physical activity was 1597.07 ± 924.47 min/week.

C3 showed the lowest observed mean levels of walking (718.13 ± 487.92 min/week), moderate-intensity physical activity (567.10 ± 380.48 min/week), and vigorous-intensity physical activity (423.48 ± 230.57 min/week). Sedentary time in C3 (2773.30 ± 1302.75 min/week) was similar in magnitude to that observed in C1, indicating that the defining feature of C3 was comparatively lower physical activity rather than markedly elevated sedentary time. The distributions of the four clustering variables are presented in Table 2.

### 3.3. Crude and Sex- and School-Type-Adjusted Associations with C2 Versus C1

In crude logistic regression analyses comparing C2 with C1, sex was not associated with profile membership (boys versus girls: OR = 1.16, 95% CI: 0.90–1.51, *p* = 0.259). Compared with students attending general secondary schools, students attending technical secondary schools had higher odds of C2 membership (OR = 1.37, 95% CI: 1.01–1.86, *p* = 0.040), whereas no statistically significant association was observed for stage I vocational school students (OR = 1.18, 95% CI: 0.81–1.69, *p* = 0.383).

Among the lifestyle correlates, school-day sleep duration of ≥9 h/day was associated with higher odds of C2 membership compared with 7–8 h/day in the crude analysis (OR = 2.05, 95% CI: 1.16–3.61, *p* = 0.013). Weekend sleep duration of ≥9 h/day was also associated with C2 membership (OR = 1.55, 95% CI: 1.15–2.09, *p* = 0.004). Weekend sleep duration of <7 h/day (*p* = 0.051) and irregular eating patterns (*p* = 0.058) did not reach the predefined threshold for statistical significance in the crude analyses.

After adjustment for sex and school type, four associations reached statistical significance. Participants reporting irregular eating patterns had higher odds of C2 membership than those reporting regular eating patterns (OR = 1.73, 95% CI: 1.05–2.84, *p* = 0.030). School-day sleep duration of ≥9 h/day was associated with higher odds of C2 membership compared with 7–8 h/day (OR = 1.98, 95% CI: 1.11–3.54, *p* = 0.022). For weekend sleep, both <7 h/day (OR = 1.70, 95% CI: 1.02–2.82, *p* = 0.041) and ≥9 h/day (OR = 1.59, 95% CI: 1.16–2.16, *p* = 0.027) were associated with higher odds of C2 membership relative to the 7–8 h/day reference category.

No statistically significant adjusted associations with C2 membership were observed for the dietary indices, Internet-use-related risk, TV/computer screen time, between-meal snacking, eating meals away from home, any reported alcohol consumption, any reported energy drink consumption, current smoking, or any of the four HBI domain scores (all *p* > 0.05). Statistically significant adjusted associations are summarised in Table 3.

Complete crude and adjusted results for the C2-versus-C1 contrast are provided in Table S2A,B.

### 3.4. Crude and Sex- and School-Type-Adjusted Associations with C3 Versus C1

In crude logistic regression analyses comparing C3 with C1, sex was not associated with profile membership (boys versus girls: OR = 1.12, 95% CI: 0.94–1.33, *p* = 0.209). Students attending stage I vocational schools had higher odds of C3 membership than students attending general secondary schools (OR = 1.34, 95% CI: 1.05–1.69, *p* = 0.018), whereas no statistically significant association was observed for technical secondary school students (OR = 1.04, 95% CI: 0.84–1.28, *p* = 0.722). None of the examined lifestyle candidate correlates showed a statistically significant crude association with C3 versus C1 membership.

After adjustment for sex and school type, none of the examined lifestyle candidate correlates reached statistical significance (all *p* > 0.05). No statistically significant adjusted associations were observed for the dietary indices, Internet-use-related risk, TV/computer screen time, regular eating patterns, between-meal snacking, eating meals away from home, school-day or weekend sleep duration, any reported alcohol consumption, any reported energy drink consumption, current smoking, or any of the four HBI domain scores.

**Figure 1:**
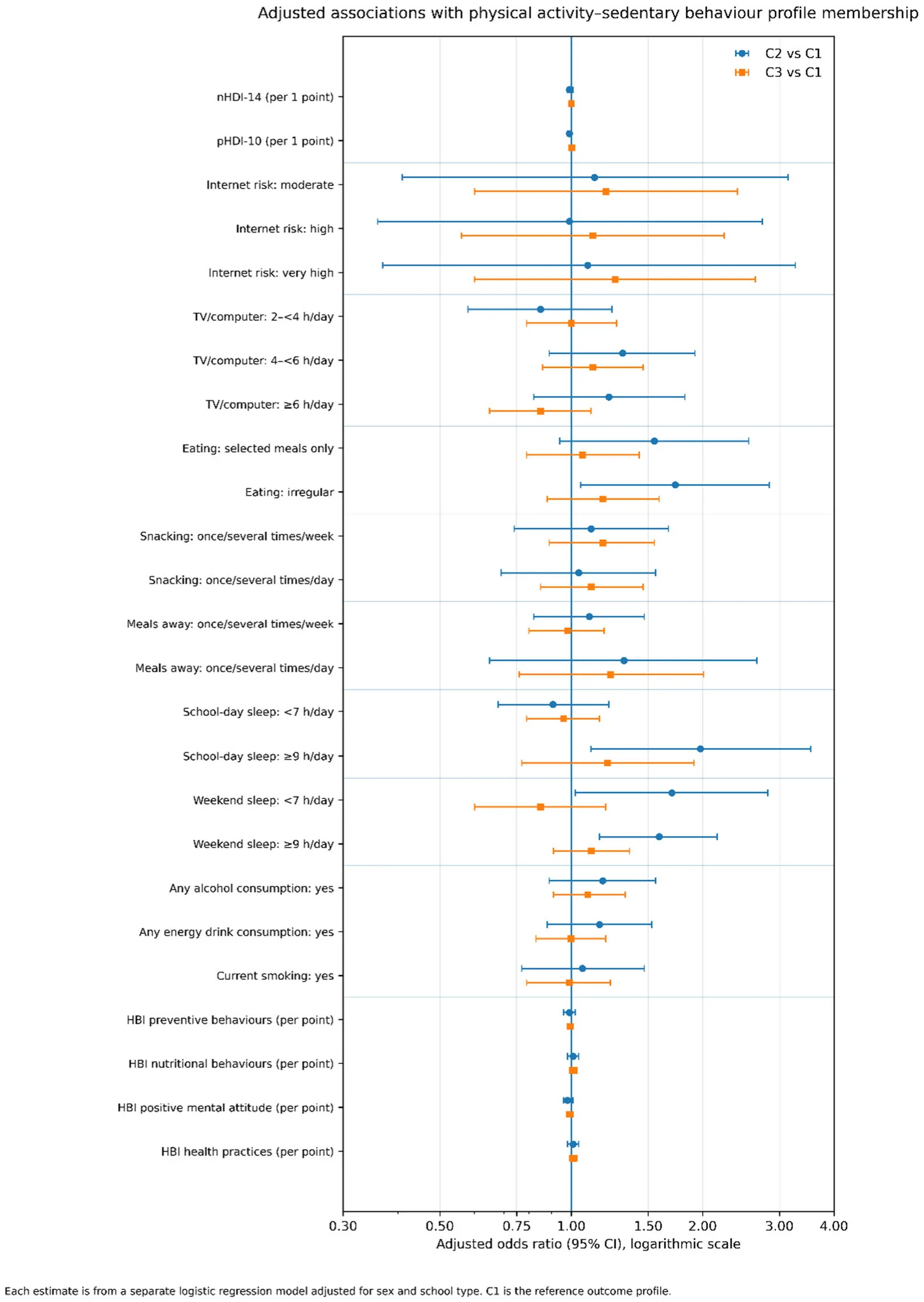
Sex- and school-type-adjusted associations with physical activity–sedentary behaviour profile membership. Points represent adjusted odds ratios (ORs), and horizontal lines represent 95% confidence intervals (95% CIs). Each candidate correlate was evaluated in a separate binary logistic regression model adjusted for sex and school type. C1 (walking-dominant activity profile) was the reference outcome category. C2 represents the high moderate-to-vigorous activity/lower sedentary profile, and C3 represents the lower physical activity profile. The vertical line at OR = 1.0 indicates the null association.

Overall, statistically significant adjusted associations with selected eating-regularity and sleep-duration categories were observed in the C2-versus-C1 contrast, whereas no lifestyle candidate correlate reached statistical significance in the C3-versus-C1 contrast. These findings describe the pattern of statistical significance within each contrast; no formal statistical comparison of regression coefficients between the two contrasts was performed. Adjusted associations for both contrasts are visualised in Figure 1.

Complete crude and adjusted results for the C3-versus-C1 contrast are provided in Table S3A,B.

## 4. Discussion

### 4.1. Principal Findings and Contribution

The standardised clustering procedure identified three movement-behaviour profiles: a walking-dominant activity profile (C1), a high moderate-to-vigorous activity/lower sedentary profile (C2), and a lower physical activity profile (C3). Importantly, the lower physical activity profile was not characterised by markedly higher sedentary time than C1. Instead, C1 and C3 showed similar levels of sedentary time but differed substantially in walking, moderate-intensity, and vigorous-intensity physical activity. This finding reinforces the concept that physical activity and sedentary behaviour represent related but distinct behavioural dimensions and should not be interpreted as opposite ends of a single continuum.

The analyses further showed that membership in the high moderate-to-vigorous activity/lower sedentary profile was associated with selected eating and sleep characteristics. Compared with adolescents reporting regular eating patterns, those reporting irregular eating had higher odds of C2 membership. Higher odds of C2 membership were also observed for school-day sleep duration of ≥9 h/day and for both <7 h/day and ≥9 h/day of weekend sleep, relative to the 7–8 h/day reference category. In contrast, none of the examined lifestyle candidate correlates reached statistical significance in the adjusted C3-versus-C1 analyses. These findings should not be interpreted as demonstrating statistically different associations between C2 and C3, because the regression coefficients from the two contrasts were not formally compared. Rather, the two analyses showed different patterns of statistical significance within the set of lifestyle characteristics examined.

These findings extend previous person-oriented research showing that physical activity, sedentary behaviour, dietary behaviours, and other lifestyle characteristics cluster into heterogeneous configurations during adolescence [4,5]. Previous activity-related typologies have similarly identified active, inactive, sedentary, and mixed profiles, supporting the view that movement behaviours are embedded within broader behavioural contexts rather than forming a simple healthy–unhealthy gradient [6]. The present study adds to this literature in two respects. First, it shows that an empirically derived lower-activity profile may occur without a corresponding increase in sedentary time. Second, the high moderate-to-vigorous activity/lower sedentary profile was not uniformly characterised by favourable lifestyle attributes, as its membership was associated with both irregular eating and several sleep-duration categories outside the 7–8 h/day reference category. Together, these observations support a multidimensional interpretation of adolescent movement behaviour while also indicating that the lifestyle characteristics accompanying empirically derived activity profiles may be more heterogeneous than would be expected from physical activity level alone.

### 4.2. Interpretation of the Movement-Behaviour Profiles

The cluster solution provides an important perspective on the relationship between physical activity and sedentary behaviour in adolescents. C1 was characterised by the highest walking activity and relatively high moderate-intensity activity, whereas C2 combined the highest moderate- and vigorous-intensity activity with the lowest sedentary time. In contrast, C3 showed substantially lower walking, moderate-intensity, and vigorous-intensity activity than the other profiles, while its sedentary time was similar in magnitude to that observed in C1. Thus, the major distinction between C1 and C3 was the amount of physical activity rather than sedentary time.

This pattern supports the view that physical activity and sedentary behaviour should be considered related but distinct behavioural dimensions. Previous person-oriented research has identified active, inactive, sedentary, and mixed lifestyle profiles among adolescents, indicating that higher physical activity does not necessarily imply lower sedentary behaviour and, conversely, that lower physical activity does not necessarily coincide with the highest sedentary exposure [4,5,6]. Similar mixed configurations have been reported in studies describing physically active adolescents who also exhibit other potentially less favourable lifestyle characteristics [12,13,14]. The present results extend this perspective by showing that a large lower-activity profile can emerge without being characterised by markedly greater sedentary time than a more active comparison profile.

The interpretation of these clusters should nevertheless remain relative to the present sample. The labels describe the observed configuration of the four variables used in the clustering procedure rather than externally defined behavioural categories. In particular, the term “lower physical activity profile” does not imply that all adolescents in C3 met a clinical or guideline-based definition of insufficient physical activity. Likewise, the comparatively favourable movement pattern observed in C2 should not be interpreted as evidence of an overall healthy lifestyle profile, because clustering was based exclusively on sedentary time and three components of physical activity.

### 4.3. Sleep-Duration Patterns in the High Moderate-to-Vigorous Activity/Lower Sedentary Profile

Sleep duration was the behavioural domain most consistently associated with C2 membership after adjustment for sex and school type. Compared with the 7–8 h/day reference category, school-day sleep of ≥9 h/day was associated with higher odds of membership in the high moderate-to-vigorous activity/lower sedentary profile. For weekend sleep, both <7 h/day and ≥9 h/day were associated with higher odds of C2 membership. The observed pattern therefore does not suggest a simple gradient in which either shorter or longer sleep consistently accompanies higher physical activity. Rather, several sleep-duration categories outside the reference category were associated with C2 membership.

This finding is compatible with the broader literature indicating that sleep, physical activity, and sedentary behaviour are interrelated components of adolescent daily behaviour [11]. Previous studies have also shown that sleep patterns in young people are associated with other behavioural and contextual factors, including screen-based activities [15,16]. However, TV/computer screen time was not statistically associated with C2 membership after adjustment in the present study. The sleep associations observed here therefore cannot be attributed to screen exposure on the basis of these data.

Several mechanisms could potentially contribute to the observed pattern, but they remain speculative. Adolescents engaging in higher levels of moderate-to-vigorous activity may differ in school schedules, organised sport participation, training times, commuting, social activities, recovery practices, or weekend routines. Conversely, longer reported sleep in some adolescents may reflect greater recovery needs or differences in daily schedules. The present study did not assess organised sport participation, training frequency, bedtime and wake time, sleep quality, daytime napping, or social jetlag, and therefore cannot distinguish among these explanations.

Importantly, the sleep categories used in the present analyses were questionnaire-based categories rather than classifications of adequate or inadequate sleep. Accordingly, the findings should not be interpreted as indicating that either ≥9 h/day or <7 h/day caused the high-activity profile, nor that the 7–8 h/day reference category represents an optimal sleep duration for all participants. Instead, the results indicate an association between the distribution of reported sleep duration and membership in the C2 movement-behaviour profile.

### 4.4. Eating Regularity in the High Moderate-to-Vigorous Activity/Lower Sedentary Profile

Irregular eating was the only dietary characteristic that remained statistically associated with C2 membership after adjustment for sex and school type. Adolescents reporting irregular eating patterns had higher odds of belonging to C2 than those reporting regular eating patterns. In contrast, the broader pro-healthy and non-healthy dietary indices, between-meal snacking, eating meals away from home, and the Health Behaviour Inventory nutritional-behaviour domain were not statistically associated with C2 membership after adjustment.

This distinction is important because the finding should not be interpreted as evidence that adolescents in C2 had an overall less favourable diet. Rather, the association concerned the regularity of eating occasions, whereas the measures describing food-group consumption and other dietary behaviours did not show corresponding adjusted associations. Previous studies have demonstrated that dietary behaviours and physical activity can form heterogeneous lifestyle configurations in adolescents [4,5]. Polish research has also identified combined dietary–activity patterns among teenagers, including prudent-active and fast-food-sedentary configurations [19]. The present findings are consistent with the broader concept that movement behaviours may coexist with heterogeneous eating behaviours, although the specific association with meal regularity requires confirmation in other populations.

The mechanisms underlying this association cannot be determined from the present data. Higher levels of moderate-to-vigorous physical activity could coexist with irregular meal timing because of school schedules, extracurricular activities, organised sport, commuting, or other time constraints. Alternatively, both movement behaviour and meal regularity may reflect broader family, school, or socioeconomic routines that were not fully captured in the present analysis. These explanations remain hypothetical because the questionnaire assessed the regularity of eating patterns but did not provide detailed information on meal timing, skipped meals, energy intake, dietary composition at individual eating occasions, or the temporal relationship between meals and physical activity.

### 4.5. The Lower Physical Activity Profile and the Absence of Adjusted Lifestyle Associations

A notable finding of the analyses was the absence of statistically significant adjusted associations between the examined lifestyle candidate correlates and membership in C3 relative to C1. Although stage I vocational-school attendance was associated with C3 membership in the crude analysis, none of the lifestyle characteristics examined—including sleep duration, TV/computer screen time, Internet-use-related risk, dietary behaviours, alcohol or energy drink consumption, smoking, and the Health Behaviour Inventory domains—reached statistical significance after adjustment for sex and school type.

This result requires cautious interpretation. It does not demonstrate that lifestyle factors are unrelated to lower physical activity among adolescents. Rather, within the set of variables examined and using C1 as the comparison profile, the present analyses did not identify statistically significant adjusted lifestyle associations with C3 membership. Absence of statistical significance should therefore not be interpreted as evidence of absence of behavioural determinants.

One possible explanation is that C3 represented a comparatively broad and heterogeneous group. It comprised approximately 70% of the study sample, and its defining feature was relatively lower physical activity rather than an extreme pattern of sedentary behaviour. Adolescents assigned to this profile may therefore differ considerably in the individual, social, and environmental circumstances associated with their movement behaviour. Such heterogeneity could reduce the likelihood that a limited set of self-reported lifestyle variables would clearly distinguish C3 from the walking-dominant C1 profile.

Furthermore, lower physical activity may be related to factors that were not included in the present analyses. These may include access to recreational facilities, active transportation, opportunities for organised sport, school physical activity provision, family support, peer influences, neighbourhood characteristics, perceived competence, motivation, health status, and psychosocial factors. These possibilities should be examined in future studies rather than inferred from the present cross-sectional data.

The analyses do not support a simple division between one lifestyle configuration characterised by high activity and another characterised by high sedentary exposure. Instead, they suggest that comparatively lower physical activity may occur within a broad behavioural group that is not easily differentiated by the lifestyle variables assessed here.

### 4.6. Implications for Research and Health Promotion

The present findings support a multidimensional interpretation of adolescent movement behaviour. The cluster solution showed that comparatively lower physical activity did not necessarily coincide with markedly higher sedentary time, while the high moderate-to-vigorous activity/lower sedentary profile was associated with selected sleep-duration categories and irregular eating patterns. These observations suggest that physical activity volume alone may provide an incomplete characterisation of the broader behavioural context in which adolescent movement behaviours occur.

For research purposes, the findings indicate that future studies should consider physical activity, sedentary behaviour, sleep, and eating behaviours as related but non-interchangeable components of adolescent lifestyle. In particular, the associations observed for C2 suggest that highly active adolescents may still show heterogeneous patterns in other lifestyle domains. Sleep duration and meal regularity may therefore be relevant characteristics to examine alongside movement behaviour in future longitudinal and intervention studies. However, the present cross-sectional results do not establish whether these characteristics precede, result from, or share common determinants with membership in the high moderate-to-vigorous activity/lower sedentary profile.

The absence of statistically significant adjusted lifestyle associations for C3 is also informative. It suggests that the large lower physical activity profile identified in the present sample may not be adequately characterised by the individual lifestyle behaviours assessed here. Future research should therefore extend beyond behavioural self-report measures and include potential social, environmental, school-related, and psychosocial determinants of physical activity. Factors such as access to opportunities for organised activity, active transportation, family and peer support, school physical activity environments, perceived competence, and motivational characteristics may help explain heterogeneity within lower-activity adolescents.

Objective assessment should also be prioritised in future studies. Accelerometry could provide more precise estimates of physical activity intensity and sedentary time, while device-based sleep assessment could complement questionnaire-based sleep-duration categories. More comprehensive measures of digital behaviour should distinguish television and computer use from smartphone, tablet, gaming, and other screen-based activities. Repeated or longitudinal measurements would further allow examination of the stability of empirically derived profiles and of transitions between them over time.

From a health-promotion perspective, the results support caution against assuming that high physical activity necessarily corresponds to favourable behaviours across all lifestyle domains. At the same time, the present findings are insufficient to define profile-specific intervention targets. The associations observed for sleep duration and eating regularity in C2 should therefore be regarded as hypothesis-generating rather than prescriptive. For C3, the absence of adjusted lifestyle associations further argues against deriving specific preventive recommendations from the present analysis alone. A broader assessment of individual and contextual factors may be required before movement-behaviour profiles can be translated into targeted health-promotion strategies.

### 4.7. Strengths and Limitations

Several strengths of the present study should be acknowledged. First, the study included a large school-based sample of 3307 adolescents recruited using a stratified sampling approach based on school type and location. Second, the person-oriented analytical approach allowed physical activity and sedentary behaviour to be examined jointly rather than reduced to a single summary indicator. In the analysis, all clustering variables were standardised before k-means clustering, and reported physical activity durations were processed using the predefined truncation procedure. Third, the study included a broad range of lifestyle characteristics spanning sleep, digital behaviours, eating behaviours, dietary indices, substance-related behaviours, and general health behaviours. Finally, the regression strategy evaluated candidate correlates in separate models adjusted for sex and school type, without *p*-value-based pre-screening or stepwise variable selection.

Several limitations should also be considered. First, the cross-sectional design precludes conclusions about temporality or causality. The observed associations therefore describe relationships with profile membership but cannot establish whether the examined lifestyle characteristics precede, result from, or share common determinants with the movement-behaviour profiles.

Second, physical activity, sedentary time, sleep duration, dietary behaviours, and other lifestyle characteristics were based on self-report and are therefore susceptible to recall error and misclassification. Although the IPAQ processing reduced the influence of extreme reported activity values, objective measurements would provide greater precision. Future studies should therefore replicate the identified profiles using device-based measures of physical activity, sedentary behaviour, and sleep. BMI was unavailable for 423 participants (12.79%), and the mechanism of missingness was not formally assessed; however, BMI was used descriptively and was not included in either cluster derivation or the regression models.

Third, the cluster solution was exploratory and sample-dependent. Although the three-cluster solution provided a more differentiated and substantively interpretable representation of the observed movement-behaviour patterns, its average silhouette coefficient was modest (0.28) and lower than that of the two-cluster solution (0.36). The profiles should therefore be interpreted as empirical configurations rather than sharply separated behavioural categories. Cluster sizes were also markedly unequal, with C3 comprising approximately 70% of the sample, which may indicate substantial heterogeneity within the lower physical activity profile.

Fourth, some candidate measures overlapped conceptually. In particular, the dietary indices, individual dietary behaviours, and the nutritional domain of the Health Behaviour Inventory partly assessed related constructs. These variables were evaluated in separate regression models rather than entered simultaneously, reducing statistical collinearity but not eliminating conceptual overlap.

Fifth, participants were nested within schools, whereas within-school dependence was not explicitly modelled. Adjustment for school type does not fully account for potential correlation among students attending the same school, and this should be considered when interpreting the precision of the regression estimates.

Sixth, multiple candidate correlates and categorical contrasts were examined. Because no formal correction for multiple testing was applied, associations with *p*-values close to the conventional 0.05 threshold should be regarded as exploratory and require replication.

Seventh, the regression coefficients from the C2-versus-C1 and C3-versus-C1 analyses were not formally compared. Consequently, statistical significance in one contrast and absence of significance in another should not be interpreted as evidence that the corresponding associations differed statistically between profiles.

Finally, the study was conducted among secondary-school students from the Świętokrzyskie Voivodeship in south-central Poland. Although stratified sampling increased heterogeneity within the regional sample, the identified profiles and their accompanying characteristics may not generalise directly to adolescents in other Polish regions, other countries, different educational systems, or different socioeconomic and cultural contexts. Replication in more geographically and socially diverse populations is therefore needed.

## 5. Conclusions

The analyses identified three physical activity–sedentary behaviour profiles among adolescents: a walking-dominant activity profile, a high moderate-to-vigorous activity/lower sedentary profile, and a lower physical activity profile. The lower physical activity profile was not characterised by markedly higher sedentary time than the walking-dominant profile, supporting the view that physical activity and sedentary behaviour represent related but distinct dimensions of adolescent movement behaviour.

After adjustment for sex and school type, irregular eating patterns and selected sleep-duration categories were associated with membership in the high moderate-to-vigorous activity/lower sedentary profile, whereas no lifestyle candidate correlate reached statistical significance in the adjusted lower-activity versus walking-dominant comparison. These findings should be interpreted as exploratory patterns of association rather than evidence of causal relationships or statistically different associations between profiles. Longitudinal studies incorporating objective measures are needed to determine the stability and broader health significance of these movement-behaviour profiles.

## Figures and Tables

**Table 1:** Characteristics of the study sample and data availability.

Variable	Available N	Value	Missing, *n* (%)
Total sample	3307	3307	0 (0.00)
Sex	3307	Girls: 1727 (52.22%); boys: 1580 (47.78%)	0 (0.00)
Age, years	3307	15.89 ± 0.71; range 15–17	0 (0.00)
BMI, kg/m^2^	2884	21.58 ± 4.69	423 (12.79)
Current smoking	3130	Yes: 759 (24.25%)	177 (5.35)
Any reported alcohol consumption	3206	Yes: 1219 (38.02%)	101 (3.05)
Any reported energy drink consumption	3206	Yes: 1745 (54.43%)	101 (3.05)
School-day sleep duration	3122	<7 h/day: 1385 (44.36%)	185 (5.59)
Weekend sleep duration	3118	≥9 h/day: 1722 (55.23%)	189 (5.72)
Between-meal snacking	3186	Once/several times/day: 1425 (44.73%)	121 (3.66)
Eating meals away from home	3108	Once/several times/day: 137 (4.41%)	199 (6.02)
TV/computer screen time	3140	≥6 h/day: 591 (18.82%)	167 (5.05)
Internet-use-related risk	3307	High or very high: 1795 (54.28%)	0 (0.00)
HBI: nutritional behaviours	3307	17.11 ± 4.69; 17 (IQR 6)	0 (0.00)
HBI: preventive behaviours	3307	16.98 ± 4.85; 17 (IQR 6)	0 (0.00)
HBI: positive mental attitude	3307	18.43 ± 5.02; 19 (IQR 7)	0 (0.00)
HBI: health practices	3307	18.14 ± 4.42; 18 (IQR 6)	0 (0.00)

Notes: Values are presented as *n* (%) or mean ± SD; median (IQR), as appropriate. Percentages for categorical variables were calculated using the number of participants with available data for the respective variable as the denominator. Missing percentages were calculated relative to the total study sample (N = 3307). BMI, body mass index; HBI, Health Behaviour Inventory; IQR, interquartile range; SD, standard deviation.

**Table 2:** Movement-behaviour characteristics of the three physical activity–sedentary behaviour profiles.

Variable, Min/Week	C1: Walking-Dominant Activity Profile (*n* = 645; 19.50%)	C2: High Moderate-to-Vigorous Activity/Lower Sedentary Profile (*n* = 354; 10.71%)	C3: Lower Physical Activity Profile (*n* = 2308; 69.80%)	*p*-Value
Sedentary behaviour	2785.78 ± 1354.91	2297.94 ± 383.83	2773.30 ± 1302.75	0.001
Walking PA	1848.20 ± 831.61	1597.07 ± 924.47	718.13 ± 487.92	0.001
Moderate-intensity PA	1714.70 ± 819.06	1951.07 ± 1233.05	567.10 ± 380.48	0.001
Vigorous-intensity PA	507.98 ± 229.37	1425.51 ± 383.83	423.48 ± 230.57	0.001

Notes: Values are mean ± SD. *p*-values are from global Kruskal–Wallis tests. No post hoc pairwise comparisons were performed; therefore, the *p*-values indicate an overall difference among the three profiles but do not identify which specific profile pairs differed statistically. PA, physical activity; SD, standard deviation.

**Table 3:** Statistically significant sex- and school-type-adjusted associations with C2 versus C1 profile membership.

Contrast	Candidate Correlate	Adjusted OR	95% CI	*p*-Value
C2 vs. C1	Irregular eating patterns (ref.: regular eating)	1.73	1.05–2.84	0.030
C2 vs. C1	School-day sleep ≥ 9 h/day (ref.: 7–8 h/day)	1.98	1.11–3.54	0.022
C2 vs. C1	Weekend sleep < 7 h/day (ref.: 7–8 h/day)	1.70	1.02–2.82	0.041
C2 vs. C1	Weekend sleep ≥ 9 h/day (ref.: 7–8 h/day)	1.59	1.16–2.16	0.027

Notes: Each candidate correlate was evaluated in a separate binary logistic regression model adjusted for sex and school type. C1 (walking-dominant activity profile) was the reference outcome category. No lifestyle candidate correlate reached statistical significance in the adjusted C3-versus-C1 analyses. CI, confidence interval; OR, odds ratio.

## Data Availability

The data are not publicly available due to privacy and ethical restrictions related to research involving minors. Requests for access may be directed to the corresponding author and will be considered in accordance with the participants’ consent and applicable institutional requirements.

## Supplementary Materials

The following supporting information can be downloaded at: https://www.mdpi.com/article/10.3390/nu18172798/s1, Table S1: Operational definitions, coding, and reference categories used in the revised regression analyses; Table S2A: Crude associations of the adjustment variables with C2 versus C1 profile membership; Table S2B: Crude and sex- and school-type-adjusted associations of candidate lifestyle correlates with C2 versus C1 profile membership; Table S3A: Crude associations of the adjustment variables with C3 versus C1 profile membership; Table S3B: Crude and sex- and school-type-adjusted associations of candidate lifestyle correlates with C3 versus C1 profile membership.

## References

[1] World Health Organization (2020). WHO Guidelines on Physical Activity and Sedentary Behaviour.

[2] Tremblay M.S., LeBlanc A.G., Kho M.E., Saunders T.J., Larouche R., Colley R.C., Goldfield G., Gorber S.C. (2011). Systematic review of sedentary behaviour and health indicators in school-aged children and youth. Int. J. Behav. Nutr. Phys. Act..

[3] Carson V., Hunter S., Kuzik N., Gray C.E., Poitras V.J., Chaput J.-P., Saunders T.J., Katzmarzyk P.T., Okely A.D., Gorber S.C. (2016). Systematic review of sedentary behaviour and health indicators in school-aged children and youth: An update. Appl. Physiol. Nutr. Metab..

[4] Leech R.M., McNaughton S.A., Timperio A. (2014). The clustering of diet, physical activity and sedentary behavior in children and adolescents: A review. Int. J. Behav. Nutr. Phys. Act..

[5] Alosaimi N., Sherar L.B., Griffiths P., Hamer M., Pearson N. (2024). Clusters of diet, physical activity, screen-time and sleep among adolescents and associations with 3-year change in indicators of adiposity. PLoS ONE.

[6] Parker K.E., Salmon J., Costigan S.A., Villanueva K., Brown H.L., Timperio A. (2019). Activity-related behavior typologies in youth: A systematic review. Int. J. Behav. Nutr. Phys. Act..

[7] Miranda V.P.N., Coimbra D.R., Bastos R.R., Miranda M.V., dos Santos Amorim P.R. (2021). Use of latent class analysis as a method of assessing the physical activity level, sedentary behavior and nutritional habit in the adolescents’ lifestyle: A scoping review. PLoS ONE.

[8] Porcu M., Giambona F. (2017). Introduction to latent class analysis with applications. J. Early Adolesc..

[9] Ottevaere C., Huybrechts I., Benser J., De Bourdeaudhuij I., Cuenca-Garcia M., Dallongeville J., Zaccaria M., Gottrand F., Kersting M., Rey-López J.P. (2011). Clustering patterns of physical activity, sedentary and dietary behavior among European adolescents: The HELENA study. BMC Public Health.

[10] Cabanas-Sánchez V., Martínez-Gómez D., Izquierdo-Gómez R., Segura-Jiménez V., Castro-Piñero J., Veiga O.L. (2018). Association between clustering of lifestyle behaviors and health-related physical fitness in youth: The UP&DOWN study. J. Pediatr..

[11] Kim Y., Umeda M., Lochbaum M., Stegemeier S. (2016). Physical activity, screen-based sedentary behavior, and sleep duration in adolescents: Youth Risk Behavior Survey, 2011–2013. Prev. Chronic Dis..

[12] Seghers J., Rutten C. (2010). Clustering of multiple lifestyle behaviours and its relationship with weight status and cardiorespiratory fitness in a sample of Flemish 11- to 12-year-olds. Public Health Nutr..

[13] Turner K., Dwyer J.J.M., Edwards A.M., Allison K.R. (2011). Clustering of specific health-related behaviours among Toronto adolescents. Can. J. Diet. Pract. Res..

[14] Ahmad K., Keramat S.A., Ormsby G.M., Kabir E., Khanam R. (2023). Clustering of lifestyle and health behaviours in Australian adolescents and associations with obesity, self-rated health and quality of life. BMC Public Health.

[15] Hale L., Guan S. (2015). Screen time and sleep among school-aged children and adolescents: A systematic literature review. Sleep Med. Rev..

[16] Carter B., Rees P., Hale L., Bhattacharjee D., Paradkar M.S. (2016). Association between portable screen-based media device access or use and sleep outcomes: A systematic review and meta-analysis. JAMA Pediatr..

[17] Alimoradi Z., Lin C.-Y., Broström A., Bülow P.H., Bajalan Z., Griffiths M.D., Ohayon M.M., Pakpour A.H. (2019). Internet addiction and sleep problems: A systematic review and meta-analysis. Sleep Med. Rev..

[18] Kokka I., Mourikis I., Nicolaides N.C., Darviri C., Chrousos G.P., Kanaka-Gantenbein C., Bacopoulou F. (2021). Exploring the effects of problematic Internet use on adolescent sleep: A systematic review. Int. J. Environ. Res. Public Health.

[19] Wadolowska L., Hamulka J., Kowalkowska J., Kostecka M., Wadolowska K., Biezanowska-Kopec R., Czarniecka-Skubina E., Kozirok W., Piotrowska A. (2018). Prudent-active and fast-food-sedentary dietary-lifestyle patterns: The association with adiposity, nutrition knowledge and sociodemographic factors in Polish teenagers—The ABC of Healthy Eating Project. Nutrients.

[20] Larson N., DeWolfe J., Story M., Neumark-Sztainer D. (2014). Adolescent consumption of sports and energy drinks: Linkages to higher physical activity, unhealthy beverage patterns, cigarette smoking, and screen media use. J. Nutr. Educ. Behav..

[21] Granda D., Surała O., Malczewska-Lenczowska J., Szczepańska B., Pastuszak A., Sarnecki R. (2024). Energy drink consumption among physically active Polish adolescents: Gender- and age-specific public health issue. Int. J. Public Health.

[22] Craig C.L., Marshall A.L., Sjöström M., Bauman A.E., Booth M.L., Ainsworth B.E., Pratt M., Ekelund U.L., Yngve A., Sallis J.F. (2003). International Physical Activity Questionnaire: 12-country reliability and validity. Med. Sci. Sports Exerc..

[23] Kowalkowska J., Wadolowska L., Czarnocinska J., Czlapka-Matyasik M., Galinski G., Jezewska-Zychowicz M., Bronkowska M., Dlugosz A., Loboda D., Wyka J. (2018). Reproducibility of a Questionnaire for Dietary Habits, Lifestyle and Nutrition Knowledge Assessment (KomPAN) in Polish Adolescents and Adults. Nutrients.

[24] Poprawa R. (2011). Test problematycznego używania Internetu. Adaptacja i ocena psychometryczna Internet Addiction Test K. Young Przegląd Psychol..

[25] Juczyński Z. (2012). Narzędzia Pomiaru w Promocji i Psychologii Zdrowia.

