# Supplementary material for: Physical Activity–Sedentary Behaviour Profiles and Associated Lifestyle Characteristics in Adolescents: A Cross-Sectional Study
Source: Nutrients. 2026 Aug 27;18(17):2798. doi: 10.3390/nu18172798 (PMC13567621; doi:10.3390/nu18172798)
Supplement: Supplementary file 1 [file nutrients-18-02798-s001.zip › nutrients-4499204-supplementary.pdf]

Supplementary Materials

S1. Operational Definitions and Coding

Table S1. Operational definitions, coding, and reference categories used in the revised regression analyses.

Variable	Operationalisation / categories	Model coding / reference
Outcome profile	C1: walking-dominant activity profile; C2: high moderate-to-vigorous activity/lower sedentary profile; C3: lower physical activity profile	Separate binary contrasts: C2 vs C1 and C3 vs C1; C1 reference outcome
Sex	Girls / boys	Girls reference; adjustment variable
School type	General secondary / technical secondary / stage I vocational	General secondary reference; adjustment variable
nHDI-14	Non-Healthy Diet Index, 0–100	Continuous, per 1-point increase
pHDI-10	Pro-Healthy Diet Index, 0–100	Continuous, per 1-point increase
Internet-use-related risk	Low/very low; moderate; high; very high	Low/very low reference
TV/computer screen time	<2; ≥ 2 to <4; ≥ 4 to <6; ≥ 6 h/day	<2 h/day reference
Regular eating patterns	Regular; regular only for selected meals; irregular	Regular reference
Between-meal snacking	Never/1–3 times/month; once/several times/week; once/several times/day	Never/1–3 times/month reference
Eating meals away from home	Never/1–3 times/month; once/several times/week; once/several times/day	Never/1–3 times/month reference
School-day sleep duration	<7; 7–8; ≥ 9 h/day	7–8 h/day reference
Weekend sleep duration	<7; 7–8; ≥ 9 h/day	7–8 h/day reference
Any reported alcohol consumption	Any reported consumption / none	None reference
Any reported energy drink consumption	Any reported consumption / none	None reference
Current smoking	Yes / no	No reference
HBI preventive behaviours	Six-item domain score, 6–30	Continuous, per 1-point increase
HBI nutritional behaviours	Six-item domain score, 6–30	Continuous, per 1-point increase
HBI positive mental attitude	Six-item domain score, 6–30	Continuous, per 1-point increase
HBI health practices	Six-item domain score, 6–30	Continuous, per 1-point increase

Notes: Each lifestyle candidate correlate was evaluated in a separate crude binary logistic regression model and in a separate model adjusted for sex and school type. No p-value-based pre-screening or stepwise variable selection was applied. The WHO physical activity recommendation variable was excluded because it overlapped conceptually with the movement variables used to derive the profile outcome. HBI, Health Behaviour Inventory; nHDI, Non-Healthy Diet Index; pHDI, Pro-Healthy Diet Index.

S2. Logistic Regression Results: C2 versus C1

Table S2A. Crude associations of the adjustment variables with C2 versus C1 profile membership.

Adjustment variable / contrast	Crude OR (95% CI)	p-value
Sex: male (ref. girl)	1.16 (0.90–1.51)	0.259
School type: stage I vocational (ref. general secondary)	1.18 (0.81–1.69)	0.383
School type: technical secondary (ref. general secondary)	1.37 (1.01–1.86)	0.040

Table S2B. Crude and sex- and school-type-adjusted associations of candidate lifestyle correlates with C2 versus C1 profile membership.

Candidate correlate / contrast	Crude OR (95% CI)	p-value	Adjusted OR (95% CI)	p-value
nHDI-14, per 1 point	0.99 (0.98–1.01)	0.229	0.99 (0.98–1.01)	0.263
pHDI-10, per 1 point	0.99 (0.97–1.001)	0.068	0.99 (0.98–1.001)	0.074
Internet risk: moderate (ref. low/very low)	0.95 (0.39–2.35)	0.918	1.13 (0.41–3.14)	0.816
Internet risk: high (ref. low/very low)	0.83 (0.34–2.05)	0.689	0.99 (0.36–2.74)	0.980
Internet risk: very high (ref. low/very low)	0.88 (0.33–2.34)	0.796	1.09 (0.37–3.26)	0.875
TV/computer: ≥2 to <4 h/day (ref. <2)	0.90 (0.63–1.30)	0.574	0.85 (0.58–1.24)	0.400
TV/computer: ≥4 to <6 h/day (ref. <2)	1.25 (0.86–1.81)	0.240	1.31 (0.89–1.92)	0.171
TV/computer: ≥6 h/day (ref. <2)	1.23 (0.84–1.79)	0.291	1.22 (0.82–1.82)	0.318
Eating: selected meals only (ref. regular)	1.48 (0.92–2.38)	0.106	1.55 (0.94–2.55)	0.086
Eating: irregular (ref. regular)	1.58 (0.98–2.55)	0.058	1.73 (1.05–2.84)	0.030
Snacking: once/several times/week (ref. never/1–3 times/month)	0.98 (0.66–1.45)	0.909	1.11 (0.74–1.67)	0.625
Snacking: once/several times/day (ref. never/1–3 times/month)	0.99 (0.62–1.34)	0.630	1.04 (0.69–1.56)	0.862
Meals away: once/several times/week (ref. never/1–3 times/month)	1.05 (0.79–1.39)	0.744	1.10 (0.82–1.47)	0.532
Meals away: once/several times/day (ref. never/1–3 times/month)	1.17 (0.59–2.33)	0.648	1.32 (0.65–2.66)	0.441
School-day sleep: <7 h/day (ref. 7–8)	0.92 (0.70–1.22)	0.578	0.908 (0.68–1.22)	0.524
School-day sleep: ≥9 h/day (ref. 7–8)	2.05 (1.16–3.61)	0.013	1.976 (1.11–3.54)	0.022
Weekend sleep: <7 h/day (ref. 7–8)	1.62 (0.99–2.62)	0.051	1.70 (1.02–2.82)	0.041
Weekend sleep: ≥9 h/day (ref. 7–8)	1.55 (1.15–2.09)	0.004	1.59 (1.16–2.16)	0.027
Any reported alcohol consumption: yes	1.15 (0.88–1.51)	0.340	1.18 (0.89–1.56)	0.243
Any reported energy drink consumption: yes	1.19 (0.91–1.56)	0.187	1.16 (0.88–1.53)	0.287
Current smoking: yes	1.13 (0.83–1.54)	0.427	1.06 (0.77–1.47)	0.706
HBI preventive behaviours, per point	0.99 (0.96–1.02)	0.425	0.99 (0.96–1.02)	0.423
HBI nutritional behaviours, per point	1.01 (0.98–1.03)	0.828	1.01 (0.98–1.04)	0.716
HBI positive mental attitude, per point	0.99 (0.96–1.01)	0.394	0.98 (0.96–1.01)	0.193
HBI health practices, per point	1.01 (0.98–1.04)	0.582	1.01 (0.98–1.04)	0.369

S3. Logistic Regression Results: C3 versus C1

Table S3A. Crude associations of the adjustment variables with C3 versus C1 profile membership.

Adjustment variable / contrast	Crude OR (95% CI)	p-value
Sex: male (ref. girl)	1.12 (0.94–1.33)	0.209
School type: stage I vocational (ref. general secondary)	1.34 (1.05–1.69)	0.018
School type: technical secondary (ref. general secondary)	1.04 (0.84–1.28)	0.722

Table S3B. Crude and sex- and school-type-adjusted associations of candidate lifestyle correlates with C3 versus C1 profile membership.

Candidate correlate / contrast	Crude OR (95% CI)	p-value	Adjusted OR (95% CI)	p-value
nHDI-14, per 1 point	1.00 (0.99–1.01)	0.675	1.00 (0.99–1.01)	0.956
pHDI-10, per 1 point	1.00 (0.995–1.001)	0.562	1.002 (0.99–1.01)	0.688
Internet risk: moderate (ref. low/very low)	1.16 (0.61–2.20)	0.643	1.20 (0.60–2.40)	0.601
Internet risk: high (ref. low/very low)	1.10 (0.58–2.07)	0.780	1.12 (0.56–2.24)	0.746
Internet risk: very high (ref. low/very low)	1.23 (0.62–2.43)	0.559	1.26 (0.60–2.64)	0.539
TV/computer: ≥2 to <4 h/day (ref. <2)	1.02 (0.80–1.28)	0.893	1.00 (0.79–1.27)	0.984
TV/computer: ≥4 to <6 h/day (ref. <2)	1.06 (0.83–1.36)	0.642	1.12 (0.86–1.46)	0.390
TV/computer: ≥6 h/day (ref. <2)	0.88 (0.68–1.14)	0.331	0.85 (0.65–1.11)	0.224
Eating: selected meals only (ref. regular)	0.98 (0.74–1.31)	0.912	1.06 (0.79–1.43)	0.702
Eating: irregular (ref. regular)	1.11 (0.83–1.48)	0.488	1.18 (0.88–1.59)	0.275
Snacking: once/several times/week (ref. never/1–3 times/month)	1.162 (0.89–1.52)	0.273	1.18 (0.89–1.55)	0.248
Snacking: once/several times/day (ref. never/1–3 times/month)	1.049 (0.81–1.36)	0.721	1.11 (0.85–1.46)	0.444
Meals away: once/several times/week (ref. never/1–3 times/month)	0.96 (0.80–1.17)	0.709	0.98 (0.80–1.19)	0.831
Meals away: once/several times/day (ref. never/1–3 times/month)	1.23 (0.77–1.96)	0.392	1.23 (0.76–2.01)	0.404
School-day sleep: <7 h/day (ref. 7–8)	0.97 (0.81–1.17)	0.744	0.96 (0.79–1.16)	0.640
School-day sleep: ≥9 h/day (ref. 7–8)	1.23 (0.79–1.92)	0.363	1.21 (0.77–1.91)	0.410
Weekend sleep: <7 h/day (ref. 7–8)	0.84 (0.60–1.16)	0.291	0.85 (0.60–1.20)	0.359
Weekend sleep: ≥9 h/day (ref. 7–8)	0.96 (0.70–1.32)	0.807	1.11 (0.91–1.36)	0.298
Any reported alcohol consumption: yes	1.13 (0.94–1.35)	0.201	1.09 (0.91–1.33)	0.323
Any reported energy drink consumption: yes	1.04 (0.87–1.24)	0.681	0.997 (0.83–1.20)	0.971
Current smoking: yes	1.03 (0.83–1.27)	0.798	0.99 (0.79–1.23)	0.920
HBI preventive behaviours, per point	0.99 (0.97–1.01)	0.371	0.995 (0.98–1.01)	0.571
HBI nutritional behaviours, per point	1.01 (0.99–1.03)	0.371	1.01 (0.99–1.03)	0.281
HBI positive mental attitude, per point	0.996 (0.979–1.01)	0.666	0.99 (0.98–1.01)	0.525
HBI health practices, per point	1.004 (0.98–1.02)	0.723	1.01 (0.99–1.03)	0.313
